# Challenges of Data Availability and Use in Conducting Health-EDRM Research in a Post-COVID-19 World

**DOI:** 10.3390/ijerph19073917

**Published:** 2022-03-25

**Authors:** Emily Ying Yang Chan, Debarati Guha-Sapir, Caroline Dubois, Rajib Shaw, Chi Sing Wong

**Affiliations:** 1 Collaborating Centre for Oxford University and CUHK for Disaster and Medical Humanitarian Response, Hong Kong, China; cswong@cuhk.edu.hk; 2 Nuffield Department of Medicine, University of Oxford, Oxford OX3 7BN, UK; 3 JC School of Public Health and Primary Care, Faculty of Medicine, The Chinese University of Hong Kong, Hong Kong, China; caroline.dubois@gxfoundation.hk; 4 GX Foundation, Hong Kong, China; 5 Accident & Emergency Medicine Academic Unit, The Chinese University of Hong Kong, Prince of Wales Hospital, Hong Kong, China; 6 Centre for Research on the Epidemiology of Disasters, Institute of Health and Society, Université Catholique de Louvain, 1200 Brussels, Belgium; debarati.guha@uclouvain.be; 7 Center for Humanitarian Health, Johns Hopkins University, Baltimore, MD 21205, USA; 8 Graduate School of Media and Governance, Keio University, Fujisawa 252-0882, Japan; shaw@sfc.keio.ac.jp

**Keywords:** health-EDRM, data use, data availability, data management, COVID-19, biological hazards, disasters, natural disasters

## Abstract

Disasters disrupt communication channels, infrastructure, and overburden health systems. This creates unique challenges to the functionality of surveillance tools, data collection systems, and information sharing platforms. The WHO Health Emergency and Disaster Risk Management (Health-EDRM) framework highlights the need for appropriate data collection, data interpretation, and data use from individual, community, and global levels. The COVID-19 crisis has evolved the way hazards and risks are viewed. No longer as a linear event but as a protracted hazard, with cascading and compound risks that affect communities facing complex risks such as climate-related disasters or urban growth. The large-scale disruptions of COVID-19 show that disaster data must evolve beyond mortality and frequency of events, in order to encompass the impact on the livelihood of communities, differentiated between population groups. This includes relative economic losses and psychosocial damage. COVID-19 has created a global opportunity to review how the scientific community classifies data, and how comparable indicators are selected to inform evidence-based resilience building and emergency preparedness. A shift into microlevel data, and regional-level information sharing is necessary to tailor community-level interventions for risk mitigation and disaster preparedness. Real-time data sharing, open governance, cross-organisational, and inter-platform collaboration are necessary not just in Health-EDRM and control of biological hazards, but for all natural hazards and man-made disasters.

## 1. Introduction

Data availability and use are necessary considerations in the improvement of Health Emergency and Disaster Risk Management (Health-EDRM). Data are used to support disaster mitigation through top-down government efforts and bottom-up community resilience building. Good quality data aid investment in research; evidence-based policies; and design of impactful programmes. The Health-EDRM framework provides a common language and comprehensive mechanism to enable actors in health and other sectors to reduce health risks and consequences of natural and man-made disasters [1]. The framework reinforces the need to generate data at a global scale, and also at individual and household levels. This differentiation is important because disasters follow a non-linear transition, with cascading compound risks. Differentiation is required to develop adaptable, targeted, and nuanced risk prevention and risk mitigation measures for communities.

## 2. Materials and Methods

This paper summarises the discussions of the fourth instalment of the CCOUC 10th Anniversary Webinar Series in October 2021, titled “Health EDRM and Evidence Base for Emergencies and Disasters”. This is an interdisciplinary webinar series, bringing together public health researchers, data scientists, health professionals, and students under the umbrella of Health-EDRM. The session discussed pertinent issues of data use and availability in communities facing evolving vulnerabilities such as challenges brought on by the protracted COVID-19 crisis. Data-informed pandemic management in Hong Kong was used as a case study.

## 3. Results

### 3.1. Data Challenges in the Context of Health-EDRM

Disasters create unique challenges to the functionality of data systems and value of health outcome measurements. Disruption to communication channels, infrastructure, and the health system creates barriers to information sharing and governance. With the added time pressure of disasters, these multi-faceted challenges pave a way to the politicisation of data collection, the use, and reporting [2]. Furthermore, in unfamiliar settings, such as the outbreak of new and emerging infectious diseases, scientists often lack baseline data and adequate sample sizes from which to draw comparison [3].

During non-linear transition of disasters, communities are caught in the overlap between the recovery and response waves. Communities also faced other compound and cascading risks, from other natural disasters and health threats. This complex, cyclical nature of COVID-19 has presented novel challenges in health protection, response system management, and community planning, which have impeded support strategies and restoration of local capacities. As the pandemic evolved, communities shifted in risk perceptions and capacity for behaviour change, impacting the effectiveness of health prevention and promotion strategies, and decision-making on governance strategies.

The post-COVID-19 world faces a unique window of opportunity to review the scientific community’s current understandings of data. Improvement in collection mechanisms and management frameworks will reinforce data quality and usability.

### 3.2. Strength of Microlevel Data

Data are necessary and strong tools for demonstrating the magnitude of disasters to secure the necessary resources and ensure these are used equitably. Quality and autonomy are important in creating credible indicators appropriately drives policy to maximise community protection.

The most obtainable and comparable data in disasters are lives lost and number of disaster events. The macrolevel disaster-type categorisation allows for the standardisation and comparison in evidence-based preparedness planning. Approximately 44% of disaster events worldwide are flood related, but 38 and 34% of disaster-related deaths between 1970 and 2019 are associated with tropical cyclones and droughts, respectively [4]. However, data at this macrolevel do not provide a comprehensive understanding of the disaster’s impact on the surviving community. Microlevel data describe a community’s susceptibility, risks, and specific needs during a disaster. Such aspects could include relative economic loss, impact on income, property loss, psychosocial well-being, and long-term health impacts. Microlevel data are often less stable and less generalisable. However, when used in conjunction with macro data, a nuanced understanding can be drawn that informs policies to elicit sustainable behaviour change in disaster resilience.

A case-control study in India showed that children repeatedly exposed to floods in their first year of life have statistically significant higher levels of stunting and prevalence of being underweight because of long-term malnutrition, leading to anthropometric failure in children that causes life-long detriment [5]. This evidence provides justification to key policy measures for flood prevention in vulnerable communities. For example, reviewing and improving early warning systems will not only have high return on investment in terms of cost per life saved, but also significant benefit to the end-user.

### 3.3. Data Gaps

There are gaps in standardizing the calculation of disaster impact. A lack of consensus on determining relative economic loss, for example, impedes the accurate comparison of magnitude or severity. Reported economic damage is significant when there is more to damage, but does not necessarily require emergency assistance. A report mapping several European country’s sub-national-level GDP against flood risks showed that high-income areas report greater economic losses post-disaster due to the relative cost of their infrastructure, material goods, and income loss. However, these areas are also able to rebuild better. It is instead the low-income regions that require assistance post-disaster although they reflect lower absolute economic loss [6]. Quantifications can be vastly disproportionate and should be considered relative to community circumstances.

High-quality indicators are reinforced by their credibility, defensibility, confidence, and reputation, obtained through rigorous and complete collection methodology. Real-time, open data are an important source of evidence for the identification of gaps in advancing emergency and time-sensitive public health policies. The scientific community has seen this play out in real time in the case of COVID-19, which has evolved into a protracted crisis with variable and compounding risks. Microlevel data on community behaviours throughout COVID-19 have informed decision-making in high-compliance health improvement strategies for community protection. As the possibilities of open data governance shift according to the limits of technology, they should be adapted to meet the needs of community well-being and expectations of privacy [7].

### 3.4. Application of Real-Time Data Use in a Community: Hong Kong during COVID-19

A cross-sectional telephone survey in Hong Kong showed that compliance to personal hygiene practices against COVID-19 such as mask-wearing remained high across the three waves of the pandemic. However, compliance to community measures such as social distancing wavered. Having experienced the SARS outbreak in 2003, Hong Kong has relatively transparent hospital and mortality reporting systems. The survey nevertheless identified community misconceptions: in total, 24% of respondents believed that non-symptomatic individuals could not transmit the disease, while only 63.9% believed they had sufficient knowledge to manage health risks. A significant portion of the population, particularly older adults, still relied on television for updates about the pandemic, rather than social media [8]. Real-time data generated from studies such as the one above allowed health promotion practitioners and social workers with suggestions on tailoring community outreach strategies and dissemination channels to maximise community health improvement. In the same survey, low-income households were more likely to report suffering in financial health.

A follow-up cross-sectional study conducted after the city’s third wave of COVID-19 indicated an increase in population-wide fatigue for adhering to anti-pandemic behaviour and practices, although the belief in the importance of these measures with regard to disease protection did not vary [9]. Planning and policymaking over the course of a protract crisis will evolve, following real-time interval-based monitoring.

In another population-based study investigating the impact of service suspension on families with members requiring long-term care, results showed that 25.1% of respondents engaged in regular home care responsibilities, including care of people with disabilities. Among these, 20% reported previously using community services for such care. This added burden of care comes at an economic and social cost to individuals who must take time off work or adjust their habitual schedules to provide care [10]. This research from Hong Kong became the basis for a cross-organisational, multi-disciplinary commentary piece on knowledge gaps for home care, supported by experts from Geneva, the United Kingdom, and the United States [11]. The research evolved into a global-level guideline published by the World Health Organization on the importance of supporting home care during lockdown, and the need to recognise the burden of home care provision. These guidelines aimed to strengthen community health capacities and emergency response worldwide [12].

## 4. Discussion: New Initiatives in a Post-COVID-19 World

The protract crisis of COVID-19 continues to underscore the importance of data-informed resilience building and emergency preparedness. Disasters continue to exhibit compound risks and evolving needs, not simply in infectious diseases, but in natural, man-made and technological disasters. Rapid urban grown, population mobility, and climate change, will shift disaster risk profiles and resource allocation needs. The mechanisms for data collection during disasters, and the way it is communicated, must improve with the input of various sectors to accommodate this evolution so as to inform appropriate risk mitigation efforts.

The Health-EDRM framework value micro data in creating tailored strategies and planning was apparent pre-pandemic. However, novel cross-sectoral networks and technologies that have arisen following COVID-19 have shown the possibilities for building surveillance tools, warning systems, and knowledge-based interventions that monitor patterns of health risk and behaviour during a crisis. While global-level platforms for collecting and sharing macrolevel data will continue to be very important, using data from such a high level can erase nuances about population risk necessary for optimal response [2]. Regional-level sharing platforms built on new technology will become more central in improving data collection methods and the use of data in informing risk profiles and protection mechanisms. Cross-organisational involvement of non-governmental and civil society groups should also be encouraged, to integrate databases that are currently operated in silos.

## 5. Conclusions

No standalone policy benefits every population. Data should reflect community-specific knowledge, perception, attitude, and behaviour, and the microlevel evolution of these variables under a protracted crisis. Ultimately, interdisciplinary improvements in Health-EDRM research should be used to inform policy, and push disaster risk reduction, and maximise community protection. Research efforts during COVID-19 have demonstrated the possibility of collaborating on rapid information sharing, open data governance, and integration of siloed data platform to provide real-time understandings of population needs. Researchers, health professionals, policymakers, and others must be empowered to leverage this opportunity for community health improvement, not only for biological hazards, but across other natural and man-made disasters.

## Data Availability

Not applicable.
